# Bactericidal/Permeability-Increasing Protein Is an Enhancer of Bacterial Lipoprotein Recognition

**DOI:** 10.3389/fimmu.2018.02768

**Published:** 2018-12-05

**Authors:** Sigrid Bülow, Lisa Zeller, Maren Werner, Martina Toelge, Jonas Holzinger, Clemens Entzian, Thomas Schubert, Franziska Waldow, Nicolas Gisch, Sven Hammerschmidt, André Gessner

**Affiliations:** ^1^Institute of Clinical Microbiology and Hygiene, University Hospital Regensburg, Regensburg, Germany; ^2^2bind GmbH, Am Biopark, Regensburg, Germany; ^3^Division of Bioanalytical Chemistry, Priority Area Infections, Research Center Borstel, Leibniz Lung Center, Borstel, Germany; ^4^Department of Molecular Genetics and Infection Biology, Center for Functional Genomcis of Microbes, Interfaculty Institute for Genetics and Functional Genomics, University of Greifswald, Greifswald, Germany

**Keywords:** bactericidal/permeability-increasing protein, pro-inflammatory, bacterial lipoprotein, lipoteichoic acid, *Streptococcus pneumoniae*, Gram-positive, pattern recognition

## Abstract

Adequate perception of immunologically important pathogen-associated molecular patterns like lipopolysaccharide and bacterial lipoproteins is essential for efficient innate and adaptive immune responses. In the context of Gram-negative infection, bactericidal/permeability-increasing protein (BPI) neutralizes endotoxic activity of lipopolysaccharides, and thus prohibits hyperactivation. So far, no immunological function of BPI has been described in Gram-positive infections. Here, we show a significant elevation of BPI in Gram-positive meningitis and, surprisingly, a positive correlation between BPI and pro-inflammatory markers like TNFα. To clarify the underlying mechanisms, we identify BPI ligands of Gram-positive origin, specifically bacterial lipopeptides and lipoteichoic acids, and determine essential structural motifs for this interaction. Importantly, the interaction of BPI with these newly defined ligands significantly enhances the immune response in peripheral blood mononuclear cells (PBMCs) mediated by Gram-positive bacteria, and thereby ensures their sensitive perception. In conclusion, we define BPI as an immune enhancing pattern recognition molecule in Gram-positive infections.

## Introduction

Pathogen-associated molecular patterns (PAMPs) of bacterial origin like lipopolysaccharide (LPS) are recognized by innate immune cells via a variety of pattern recognition receptors. In more detail, LPS is sensed by Toll-like receptor 4 (TLR4) in cooperation with CD14 and MD2 as well as BAI1 and Caspase-4/5 (1). In Gram-positive infections, the most important PAMPs are bacterial lipopeptides and lipoproteins [bLPs; (2, 3)]. Their extracellular recognition is mediated by TLR2 (2, 3). In this process, formation of a heterodimer between TLR2 and TLR6 or TLR1 is essential for the perception of di-acylated or tri-acylated bLPs, respectively (4, 5). Additionally, the co-receptors CD14 and, in case of di-acylated bLPs, CD36 are involved and associate with the TLR2/TLR6 or TLR2/TLR1 dimer (6–8). As a soluble factor, lipopolysaccharide binding protein (LBP) also contributes to the recognition of both LPS and bLPs by transferring these PAMPs to the CD14/TLR complexes (6, 9–11). Lipoteichoic acid (LTA) is another major component of the cell wall of Gram-positive bacteria. Lipoteichoic acids (LTAs) extracted from wild-type strains were described as TLR2 ligands (10, 12, 13). Indeed, LTA binds to TLR2, but is not sufficient to initiate TLR2 heterodimerization with TLR1 or TLR6 and consecutive signaling (5). Accordingly, LTA free of bLPs does not trigger TLR2 and is overall low- or even non-immunostimulatory (2, 14, 15). The responsible structural motifs for residual immunostimulation are not completely clarified yet, but in LTA of *Streptococcus pneumoniae* (SP) these are most likely the *P*-Cho residues present in teichoic acids, which activate the lectin complement pathway via human L-ficolin (16).

Bactericidal/permeability-increasing protein (BPI) is a neutrophil-derived protein described as the prototype of the tubular-lipid binding protein (TULIP) family (17, 18). Although showing a 44% sequence identity to LBP at the amino acid level (19), BPI has been described as the natural antagonist of LBP since the interaction of BPI with its ligand LPS is strong enough to prohibit the LPS-dependent stimulation of immune cells (20–22). This function depends on the highly cationic amino-terminal half of BPI, which fully expresses the LPS-binding potential as well as its bactericidal properties toward Gram-negative bacteria seen in holo-BPI (23). Although structurally similar to the N-terminal part (24), the C-terminal part of BPI displays no bactericidal or LPS-binding activities (25). Nevertheless, it contributes to the opsonization of Gram-negative bacteria, and is responsible for the binding of BPI to peripheral blood mononuclear cells (PBMCs) via a yet unknown receptor (26–28). The functional difference between BPI and LBP depends on the carboxy-terminal half of both proteins (29) and is mainly caused by structural characteristics in this region of the protein, thereby distinguishing LBP from BPI (19). However, N-terminal parts of both proteins show high levels of structural and functional similarity (19, 29). In addition to LPS, the N-terminal half of LBP also binds LTA and bLPs (9, 10). These lipid-containing bacterial cell wall components of Gram-positive origin have not been evaluated for BPI yet, presumably because BPI is described as non-bactericidal toward Gram-positive bacteria (30, 31). Although BPI is released in both Gram-negative and -positive bacteremia and sepsis (32, 33), its physiological role in Gram-positive infection has not been addressed so far.

In the present study, we describe a significant elevation of BPI in the cerebrospinal fluid (CSF) of patients with Gram-positive bacterial meningitis. Moreover, we show specific binding of BPI to bLPs and LTA as well as BPI-mediated enhancement of the immune response toward these ligands. In conclusion, we determine that BPI is a highly conserved pattern recognition molecule contributing to the perception of bLPs, LTA, and Gram-positive bacteria in general. This novel finding contributes to the fundamental understanding of the function of this highly expressed defense protein and the mechanisms by which the immune system detects bacterial infections.

## Materials and Methods

### Reagents

Bactericidal/permeability-increasing protein (BPI) extracted from neutrophils was purchased from Athens Research and Technology [Athens, GA, USA; BPI_N(A)_] and Wieslab AB [Malmö, Sweden; BPI_N(W)_]. Tri-acylated [Pam_3_CSK_4_, (*R*)-Pam_3_CSK_4_, (*S*)-Pam_3_CSK_4_, Pam_3_CSK_4_ Fluorescein], di-acylated [(*R*)-Pam_2_CSK_4_, (*R*)-Pam${}_{2^{*}}$CSK_4_, Pam_2_CSK_4_ biotin, (*R*)-FSL-1] and mono-acylated lipopeptides (PamCSK_4_) came from EMC Microcollections (Tübingen, Germany). Ultrapure LPS and LPS biotin both from *Escherichia coli* (EC) O111:B4 (LPS EC; LPS EC biotin) as well as peptidoglycan (PGN) purified from *Staphylococcus aureus* (SA), heat-killed lysates of *E. coli* O111:B4, *Staphylococcus aureus* and *Streptococcus pneumoniae* (SP) were purchased from Invivogen (San Diego, CA, USA). CFU/ml of the lysates are indicated according to the manufacturers declaration. Diacyl-glycerol 16:0 (DAG; Avanti Polar Lipids, Alabaster, AL, USA) dissolved in chloroform was lyophilized, solubilized in PBS, and sonicated before use. Due to the heterogeneity of the LPS of *E. coli* O111:B4 (34) an average molecular weight (MW) of 15 kDa was estimated by using gel electrophoresis. This is consistent with data provided by another distributor of LPS EC (35). PBS (Dulbecco's Phosphate Buffered Saline, endotoxin tested; Sigma Aldrich, Taufkirchen, Germany) was used for storage of recombinant BPI (rBPI), generation of bacterial lysates, BPI binding assay and NanoDSF.

### Cloning, Production, and Purification of Recombinant BPI

The expression construct was obtained by combining the sequences of an N-terminal HA signal peptide, the human BPI (aa 32-487) and a C-terminal FLAG Tag by standard cloning techniques into pCR3 Vector (Invitrogen, Carlsbad, CA, USA). In brief, HEK293T cells were transfected using the calcium phosphate mediated method. The expressed protein was purified by affinity chromatography on an anti-Flag M2 (Sigma Aldrich, Taufkirchen, Germany) coupled NHS-activated HP column (GE Healthcare, Chalfont St. Giles, Great Britain) and elution was performed with PBS containing 150 μg/ml FLAG-Peptide (Sigma Aldrich, Taufkirchen, Germany). The fractions containing the recombinant protein were concentrated via a stirred ultrafiltration cell (Amicon® Ultracel PL-10, Merck Millipore, Darmstadt, Germany) and dialyzed against PBS.

### Bacterial Strains and Preparation of Bacterial Lysates

*Streptococcus pneumoniae* strains D39Δ*cps*, D39Δ*cps*Δ*lgt*, D39Δ*cps*Δ*lsp*, and D39Δ*cps*Δ*lgt*Δ*lsp* [all non-encapsulated mutants of serotype 2 wild-type D39 36, 37), as well as *S. aureus* strains 113 and 113Δ*lgt* (3; kindly provided by F. Götz, University of Tübingen, Germany] were cultured on Columbia blood agar plates. Bacterial concentrations were determined by measuring the absorbance at 600 nm. The cell suspension was washed (3,750 × *g*) and resuspended to the desired concentration. To prepare the bacterial lysates, the suspension was heated at 65°C for 30 min under gentle agitation. Lysates were tested for viability as shown in Figure S1. The protein concentrations of the bacterial lysates were determined using the Pierce BCA Protein Assay Kit (Thermo Fisher Scientific, Waltham, MA, USA) to omit differences caused by bacterial clotting or autolysis.

### Isolation of Bacterial Cell Wall Components

Extraction and purification of LTA from *S. pneumoniae* D39Δ*cps* (LTA SP) and D39Δ*cps*Δ*lgt* (LTA SPΔ*lgt*) as well as *S. aureus* 113 (LTA SA) and 113Δ*lgt* (LTA SAΔ*lgt*) were performed according to our published protocols (14). Hydrazine (N_2_H_4_) treatment of *S. pneumoniae* LTAs and preparation of the PGN-wall teichoic acid complex (WTA) after LytA treatment from *S. pneumoniae* D39Δ*cps*Δ*lgt* were performed as described (38). Average MWs chosen for calculation of LTA concentrations are based on major signals (LTA molecules with 6 repeats) in mass spectrometry [LTA SPΔ*lgt*: 8,500 Da; LTA SPΔ*lgt*-N_2_H_4_: 8,050 Da; (14)], or average chain length as determined by ^1^H NMR (LTA SA: 4,500 Da) using a published methodology (39). In contrast to the structure of the repeating units, the exact chemical composition of the pneumococcal PGN-WTA macromolecule is not known, especially with regard to the ratio of [MurNAc-GlcNAc]-units and WTA chains. Therefore, the MW of the latter cannot be determined exactly. The MW for a pneumococcal WTA chain with 6 repeats without PGN-fragments is around 7,900 Da (40). In order to ensure that the molecular concentration of the PGN-WTA complex of *S. pneumoniae* D39Δ*cps*Δ*lgt* (WTA SPΔ*lgt*) exceeded that of LTA SPΔ*lgt* in our assays, the applied molecular concentration of WTA SPΔ*lgt* was increased 2-fold to the calculated one.

*Escherichia coli* (EC) BL21 (DE3, Novagen) was grown aerobically with shaking (150 rpm) at 37°C in standard LB medium (Invitrogen, Carlsbad, CA, USA) containing 5 g/l of NaCl until an absorbance of approximately 0.8 at 600 nm was reached. LPS was extracted by a combination of hot phenol-water (41) and PCP I extraction (42). Phenol (90%) was added to reach a final concentration of 1% and the resulting suspension was shaken (150 rpm) for 1 h at 37°C. Cells were collected by centrifugation (9,000 × *g*, 20 min, 4°C) and subsequently washed three times with water (centrifugation conditions as above). The lyophilized pellet was washed with ethanol, acetone (twice), and diethyl ether, and then dried. Cells were resuspended in water (~20 mg/ml), sequentially treated for 16 h at room temperature with DNase and RNase and 8 h with proteinase K (each at ~20 μg/ml), dialyzed for 1 day at 4°C against water (three water exchanges) and lyophilized. LPS was further extracted by phenol-chloroform-petroleum ether extraction. The pellet was suspended in phenol (90%)-chloroform-petroleum ether [40–60°C, 2:5:8 (vol/vol/vol); 95 mg/ml] with an Ultra-Turrax, then the mixture was stirred for 30 min at room temperature and the supernatant was collected after centrifugation (7,650 × *g*, 20 min, 4°C). The extraction process was repeated twice and the supernatants were then combined and evaporated in a vacuum (50–55°C) until phenol crystallization began. LPS was precipitated in the cold by the addition of water (phenol concentration >80%), then samples were centrifuged (4,300 × *g*, 20 min, 20°C) and washed twice with 80% phenol and three times with acetone (centrifugation conditions as above). A final lyophilization step yielded the LPS. A small portion of this LPS was further purified by reversed phase HPLC as previously described (43) using a modified gradient: The initial solvent system (2% B) was maintained for 20 min, followed by a linear two-step gradient rising from 2 to 17% B (20–50 min) and 17 to 20% B (50–60 min). The solvent was kept for 20 min at 20% B, followed by a linear gradient rising from 20 to 27% B (80–105 min). To ensure removal of all residual components, the solvent was kept for 30 min at 27% B, followed by a linear gradient rising from 27 to 40% B (135–140 min), kept at 40% B for 10 min, raised in 10 min by a linear gradient to 100% B and maintained for 5 min. Finally, the column was re-equilibrated for 10 min to 2% B and was maintained at that level for at least 10 min before the next injection. The pool used for the experiments contained LPS with a monoisotopic molecular mass of 3,770.723 Da as its main component, which is in line with published data for *E. coli* BL21 (DE3) LPS [LPS EC BL21; LA_hexa_+Kdo_2_+Hep_3_+Hex+HexA+*P*+*P*-Etn_2_+Ara4N; 44), and a second LPS with a mass of 3,762.692 Da (Ara4N exchanged by *P*-Etn). For the injection (sample concentration, 4 mg/ml), methanol-chloroform-water [57:12:31 (vol/vol/vol)] containing 10 mM NH_4_OAc and 0.1 M Na-EDTA, pH 7 [4:1 (vol/vol)] was used.

### BPI Microtiter Binding Assay

BPI binding assays were performed using a protocol published for LBP (9) with some modifications. Streptavidin-coated 96-well plates (Nunc^TM^ Immobilizer^TM^ Streptavidin F96 clear, Thermo Fisher Scientific, Waltham, MA, USA) were coated with LPS biotin (2 μg/ml) or Pam_2_CSK_4_ biotin (1 μg/ml) in PBS overnight at 23°C with gentle agitation. After washing with assay buffer (150 mM NaCl, 50 mM HEPES, Sigma Aldrich, Taufkirchen, Germany) containing 0.01% Casein, plates were blocked with 10 g/l BSA (Sigma Aldrich, Taufkirchen, Germany) at 37°C and washed again. Thereafter, ligands were preincubated with 20 nM BPI in assay buffer containing 1 g/l BSA for 30 min and loaded onto the plates. In case of bacterial lysates, 0.05% Tween 20 was added for preincubation. After incubation for 1 h at 37°C, three washing steps were performed. Bound BPI was detected by murine anti-human BPI monoclonal antibody (Cat.-No. HM2042, Hycult Biotech, Uden, Netherlands) and HRP-conjugated rabbit anti-mouse IgG (Cat.-No. 315-035-048, Dianova, Hamburg, Germany). TMB (BD OptEIA^TM^ TMB Substrate Reagent Set, BD Biosciences, Heidelberg, Germany) was used as a substrate for peroxidase. After addition of 1 N HCl, absorbance was measured at 450 nm. Technical replicates were performed at least two times using exactly the same reagents and are not indicated for the BPI microtiter binding assay. Biological replicates shown here are experiments independently performed by using separately formulated solutions of the reagents.

### Microscale Thermophoresis Experiments

BPI_N(A)_ and rBPI were labeled with NT647 in PBS pH 7.4 (Monolith NT™ Protein Labeling Kit RED—NHS, NanoTemper Technologies, Munich, Germany). Concentration of labeled protein was determined using the NanoDrop (ThermoScientific, Wilmington, NC, USA) and Bradford assay (Promega, Mannheim, Germany). MicroScale Thermophoresis (MST) binding experiments were carried out with 5 nM labeled protein in binding buffer (10 mM HEPES pH 7.4, 150 mM NaCl, 0.1% Tween) with 0.05–1,679 nM of LPS EC or 0.196–6,436 nM of Pam_3_CSK_4_ or 0.61–20,000 nM of LTA SAΔ*lgt* and LTA SPΔ*lgt* or of >0.61–20,000 nM WTA SPΔ*lgt* at 20–40% MST power, 20% LED power in premium capillaries on a Monolith NT.115 pico device at 25°C (NanoTemper Technologies, Munich, Germany). For MST binding assays based on Pam_3_CSK_4_ Fluorescein, 20 nM of the fluorescent molecule in binding buffer (10 mM HEPES pH 7.4, 150 mM NaCl, 0.1% Tween) was supplied with 0.018–600 nM BPI_N(A)_ and measured at 40% MST power, 10% LED power in premium capillaries on a Monolith NT.115 pico device at 25°C (NanoTemper Technologies, Munich, Germany).

MST timetraces were recorded and the temperature jump or, respectively, thermophoresis was analyzed. The recorded fluorescence was plotted against the ligand concentration and curve fitting was performed with KaleidaGraph 4.5 using the dissocation constant (K_D_) fit formula derived from the law of mass action. For better comparability, binding graphs were normalized to the fraction bound by the labeled partner (0 = unbound, 1 = bound). Technical and biological replicates are defined as described for BPI microtiter binding assays.

### NanoDSF Experiments to Determine Thermal Stability

For thermal unfolding experiments, rBPI protein was diluted to a final concentration of 5 μM in PBS pH 7.4, either containing a ligand at 30 μM concentration [(*R*)-Pam_3_CSK_4_, (*R*)-FSL-1 or LPS EC] or in the absence of a ligand. For each condition, 10 μl of sample per capillary were prepared. The samples were loaded into UV capillaries and experiments were carried out using the Prometheus NT.48 (NanoTemper Technologies, Munich, Germany). The temperature gradient was set to an increase of 1°C/min with a range from 20 to 90°C. Protein unfolding was measured by detecting the temperature-dependent change in tryptophan fluorescence at emission wavelength of 350 nm (Figure 3E, upper panel). Melting temperature (T_m_) was determined by detecting the maximum of the first derivative of the fluorescence wavelength 350 nm. For this purpose, an 8th order polynomial fit was calculated for the transition region (Figure 3E, lower panel). Next, the first derivative of the fit was formed and the peak position (at T_m_) was determined. Finally, T_m_ shift (T_m_ protein alone — T_m_ protein with ligand) upon addition of ligand to the protein (in°C) was calculated. Technical and biological replicates were defined as described for BPI microtiter binding assays.

### Measurement of BPI, LPB, and Cytokines in CSF

All available leftover samples of CSF stored in the Institute of Clinical Microbiology and Hygiene, University Hospital Regensburg in the time between 2013 and 2017 and tested positive in culture and/or PCR for either *S. pneumoniae* or *Neisseria meningitidis* were analyzed. No co-infections were present as tested by culture and/or PCR. Control samples were 20 leftover samples as indicated above tested positive for enterovirus as tested by PCR. All samples were pseudonymized. BPI, LBP, and cytokines in CSF were determined using Luminex® technology (Austin, TX, USA). BPI and LBP were measured using specific antibody pairs (αBPI capture antibody 3F9 and αBPI detection antibody 4H5, Hycult Biotech, Uden, Netherlands; αLBP capture antibody biG43 and αLBP detection antibody biG412, Biometec, Greifswald, Germany). Biotinylation of the detection antibodies was performed using the Lightning-Link® Biotin Conjugation Kit (Innova Biosciences, Cambridge, UK). Cytokines were determined with the ProcartaPlex® Multiplex Immunoassay (eBioscience, Santa Clara, CA, USA).

### Isolation and Stimulation of Human Peripheral Blood Mononuclear Cells

After informed consent, blood was drawn from healthy male volunteers using heparinized blood collection tubes (Li-Heparin-Gel-Monovette, Sarstedt, Nümbrecht, Germany) and diluted in RPMI 1640 (Biochrom, Berlin, Germany) at a ratio of 1:1.5. The blood was centrifuged in leucosep tubes containing Ficoll® Paque plus (Oxford Immunotec, Abingdon, Great Britian) at 1,000 × *g* for 10 min. The interphase containing the leukocytes was collected and subsequently washed twice with RPMI 1640. The pellet was resuspended in AIM V® Medium (Thermo Fisher Scientific, Waltham, MA, USA), counted and cultivated in 96-well plates for 4 h (1 × 10^5^/100 μl). Then cells were stimulated with the indicated compounds in the presence or absence of BPI. The supernatants were collected after 18 h for determination of cytokine concentration by ELISA according to the manufacturer's instructions (OptEIA^TM^ Human TNFα ELISA Set and OptEIA^TM^ Human IL6 ELISA, BD Biosciences, Heidelberg, Germany; DuoSet® ELISA human CXCL8/IL-8, R&D, Minneapolis, MN, USA).

### Transfection and Stimulation

HEK293T cells were transfected using the calcium phosphate mediated method according to a published protocol (45). Transfections were performed using plasmid vectors for human TLR2 and human CD14 (both a kind gift of Carsten Kirschning, University of Duisburg-Essen, Germany) at concentrations of 0.4 and 0.05 μg/ml, respectively. A GFP encoding plasmid (0.4 μg/ml) was used as control. For stimulation, transfected HEK293T cells were cultured at 2 × 10^5^ cells/ml in 96-well plates and stimulated with (*R*)-Pam_3_CSK_4_ ± BPI. After 24 h, supernatants were collected and analyzed for secretion of IL-8 by ELISA (DuoSet® ELISA human CXCL8/IL-8, R&D, Minneapolis, MN, USA).

### Ethics Statement

This study was carried out in accordance with the recommendations of the Declaration of Helsinki. All donors of PBMCs gave written informed consent in accordance with the Declaration of Helsinki. Diagnostic leftover samples stored at the Institute of Clinical Microbiology and Hygiene, University Hospital Regensburg, were used for CSF analysis. The protocol for both isolation of PBMCs and CSF analysis was approved by the local ethics committee (Ethikkommision an der Universität Regensburg).

### Statistical Analysis

All analyses were performed using GraphPad Prism, version 7.01 (GraphPad Software, San Diego, CA, USA). Results are depicted as means ± standard deviation (SD) or means ± standard error of mean (SEM). Statistical tests were performed as described in the figure legends.

## Results

### BPI is Increased in Gram-Positive Meningitis

**Figure 1:**
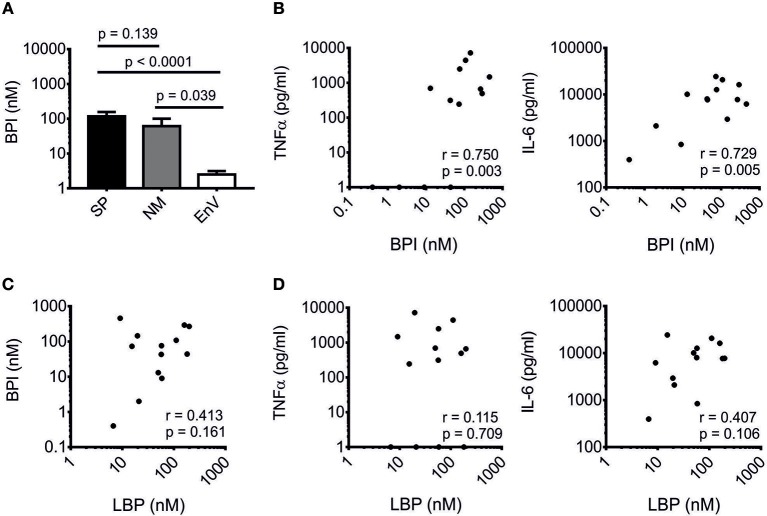
Correlation of BPI with pro-inflammatory markers in Gram-positive meningitis. BPI was measured in CSF of patients with bacterial meningitis caused by *Streptococcus pneumoniae* (SP, *n* = 13), *Neisseria meningitidis* (NM, *n* = 7) and enterovirus-infected controls (EnV, *n* = 20). **(A)** Results are expressed as means ± SEM. Statistics for comparison of the difference of the BPI level were performed with the unpaired Student's *t*-test (*p*-values are indicated). Correlation of BPI with TNFα and IL-6 **(B)** and LBP **(C)** as well as correlation of LBP with TNFα and IL-6 **(D)** are depicted. Correlation was analyzed using Pearson's correlation (*r*- and *p*-values are indicated). Logarithmic values were used for statistical testing.

Sepsis and bacteremia can be polymicrobial. In order to evaluate the increase of BPI in Gram-positive infections, we analyzed the CSF of patients with bacterial meningitis as in this specific host compartment co-infections are very unlikely and were additionally excluded by culture and/or PCR. In the case of Gram-positive meningitis, we focused on *S. pneumoniae* (*n* = 13), and in Gram-negative meningitis on *N. meningitidis* (*n* = 7). CSF of patients infected with enterovirus were used as control (*n* = 20). The protein level of BPI was significantly increased in both *S. pneumoniae* and *N. meningitidis* meningitis as compared to the control group. However, no statistically significant difference was found between *S. pneumoniae* and *N. meningitidis* meningitis (Figure 1A). Interestingly, in *S. pneumoniae* meningitis BPI positively correlated with expression levels of TNFα (*r* = 0.750, *p* = 0.003) and interleukin-6 (IL-6; *r* = 0.729, *p* = 0.005; Figure 1B). Although the pro-inflammatory role of LBP toward Gram-positive ligands has been well-established (9, 10), no correlation was found for LBP with either BPI (*r* = 0.413, *p* = 0.161; Figure 1C) or TNFα (*r* = 0.115, *p* = 0.709) and IL-6 levels (*r* = 0.407 and *p* = 0.106; Figure 1D) in *S. pneumoniae* meningitis. Contrary, in CSF of patients with meningitis caused by *N. meningitidis*, no tendency was observed for a positive correlation between BPI and either TNFα or IL-6 levels (Figure S2). In sum, BPI is elevated in *S. pneumoniae* meningitis and positively correlates with TNFα and IL-6 levels independently of LBP.

### BPI Binds to bLPs in Competition With LPS

**Figure 2:**
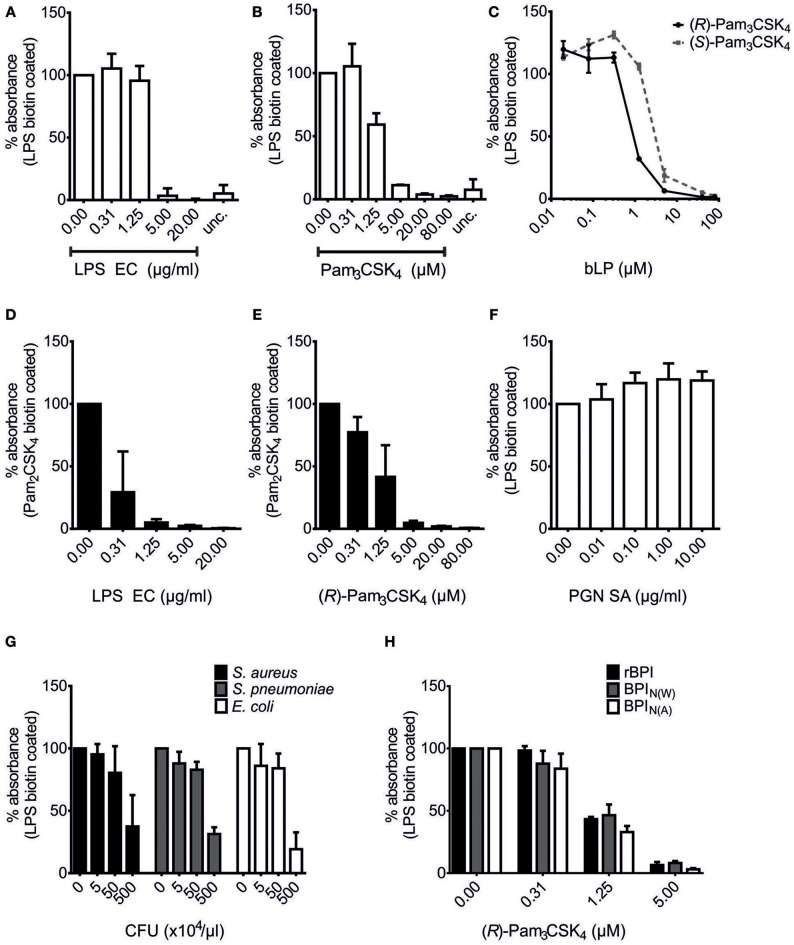
Competition of bLPs with lipopolysaccharide for binding to BPI. BPI binding assays with LPS biotin **(A–C,F–H)** or Pam_2_CSK_4_ biotin-coated plates **(D,E)**. “unc.” shows binding of rBPI in uncoated wells treated otherwise identically **(A,B)**. rBPI was pre-incubated with increasing concentrations of LPS EC **(A,D)**, the racemate Pam_3_CSK_4_ **(B)**, (*R*)-Pam_3_CSK_4_ **(C,E)** and (*S*)-Pam_3_CSK_4_ **(C)** or peptidoglycan of *S. aureus* (PGN SA; **F**). Furthermore, pre-incubations of rBPI with different heat-inactivated bacterial lysates are shown **(G)**. Preparations of rBPI and neutrophil BPI of two different sources [BPI_N(W)_ and BPI_N(A)_] were pre-incubated with (*R*)-Pam_3_CSK_4_ **(H)**. Absorbance measured at 450 nm for wells with BPI alone was set to 100% to ensure comparability between the different ligands. All results are shown as means ± SD of three biological replicates.

The N-terminal parts of BPI and LBP show high structural and functional similarity (19, 29) and are responsible for their respective potential to associate with LPS (23, 46). By comparing these two proteins, we hypothesized that TLR2-activating bLPs (2, 3) are potential ligands of BPI since they are also bound by LBP (9, 10). Binding of LBP to LPS can be inhibited with bLPs, as seen in a solid-phase LBP-binding assay (9, 10). After optimization of this assay for our purposes, binding of rBPI could be detected in wells coated with LPS biotin, but not in uncoated wells (Figure 2A). Furthermore, binding of rBPI was gradually inhibited by pre-incubation of rBPI with increasing doses of LPS of *E. coli* O111:B4 (LPS EC, Figure 2A). To avoid contamination with bLPs, commercially available ultrapure preparations of LPS were used for this purpose. Next, synthetic analogs of bLPs were tested. Interestingly, pre-incubation of rBPI with the tri-acylated lipopeptide Pam_3_CSK_4_ also inhibited binding of rBPI to LPS in a dose-dependent manner (Figure 2B). Pam_3_CSK_4_ is a racemate of (*R*)-Pam_3_CSK_4_ and (*S*)-Pam_3_CSK_4_, whereas the natural occurring enantiomer in bLPs is comprised of the (*R*)-form only. Therefore, both enantiomers were compared and a superior binding of (*R*)-Pam_3_CSK_4_ was observed (Figure 2C). When the di-acylated lipopeptide Pam_2_CSK_4_ biotin was used instead of LPS biotin for coating the plates, gradual inhibition of rBPI binding was observed for both LPS EC and (*R*)-Pam_3_CSK_4_ (Figures 2D,E). This strongly suggested that masking of LPS biotin by the bLPs can be excluded as the reason for the inhibitory effects. Moreover, the cell wall component PGN, as exemplified by PGN of *S. aureus*, did not inhibit binding of rBPI to LPS biotin, confirming the specificity of the test (Figure 2F). Interestingly, when commercially available heat-inactivated bacteria were tested, not only the Gram-negative, LPS-containing bacterium *E. coli*, but also Gram-positive, endotoxin-tested bacteria *S. aureus* and *S. pneumoniae* inhibited binding of rBPI to LPS biotin-coated plates (Figure 2G). This observation clearly implies that Gram-positive bacteria indeed contain ligands of BPI. Since rBPI was used for most of the experiments, we compared rBPI to BPI originating from neutrophils (BPI_N_) by using different commercial preparations [BPI_N(A)_, BPI_N(W)_]. All of these were similarly inhibited with respect to their binding to LPS biotin-coated wells by pre-incubation with (*R*)-Pam_3_CSK_4_ (Figure 2H).

Hence, we show the presence of BPI binding partners in Gram-positive bacteria and newly identify bacterial lipopeptides as ligands. The binding competition between LPS and bLPs suggests the presence of a common binding site for both ligands in BPI.

### BPI Binds to bLPs With High Affinity

**Figure 3:**
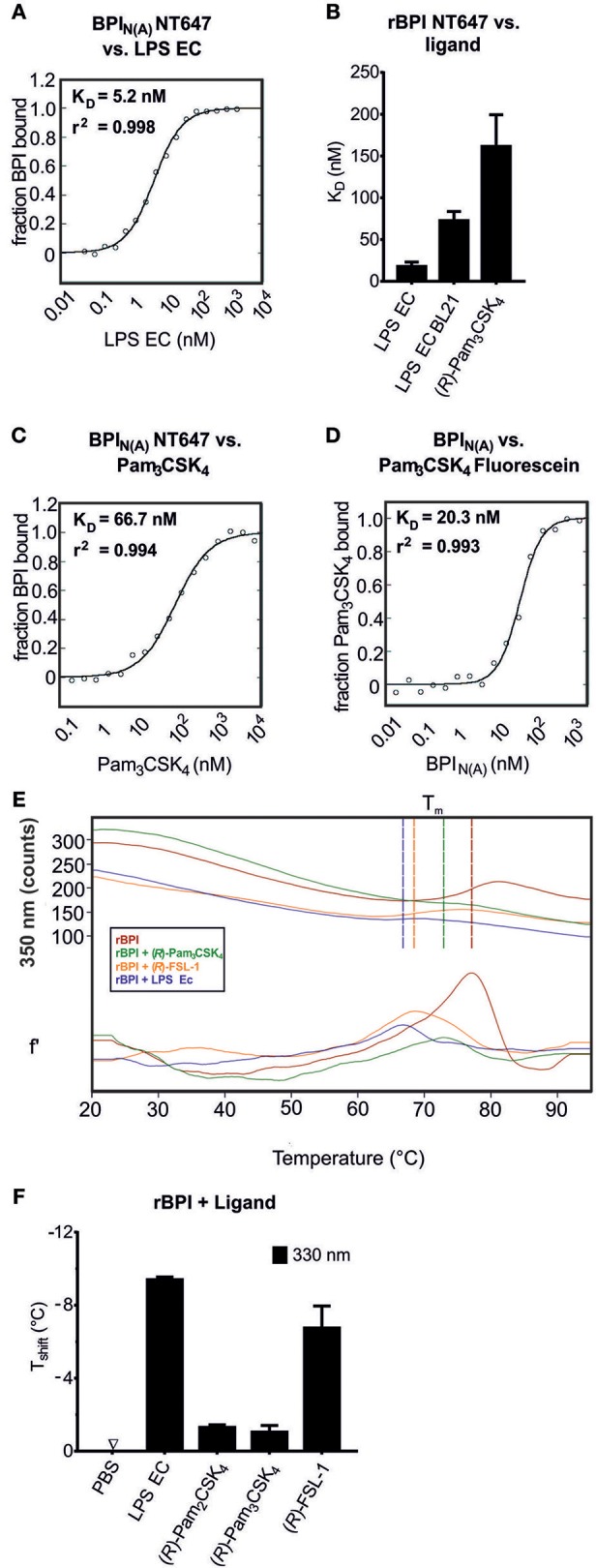
Determination of the affinity of BPI to bLPs. MST binding assay **(A–D)**. Changes in movement in MST were monitored using BPI_N(A)_ NT647 incubated with increasing concentrations of the ligands [LPS EC **(A)**, Pam_3_CSK_4_ **(C)**] or Pam_3_CSK_4_ Fluorescein incubated with increasing concentrations of BPI_N(A)_ **(D)**. The binding affinity and *r*^2^ values are indicated. The same assay using NT647-labeled rBPI was used to calculate K_D_ values for the interactions of the protein to LPS EC, LPS EC BL21, and (*R*)-Pam_3_CSK_4_ **(B)**. NanoDSF was performed for rBPI incubated with the indicated ligands **(E,F)**. Temperature-dependent change in fluorescence is indicated for the wavelength of 350 nm [**E**, upper part: absolute values, lower part: first derivate (f')]. Vertical lines indicate T_m_. The shift in melting temperature (T_shift_) caused by the ligands is shown **(F)**. Temperature shifts above 1°C are interpreted as the influence of an interaction on the thermal stability of the protein. Data represent the mean of two **(A,C,D)** or three **(B)** technical replicates or means ± SD of two biological replicates **(E,F)**.

MST was used to compare the interaction between BPI and its ligands and to determine the affinities when the binding partners are in solution. An advantage of this method is that visual control of the binding curves allows optimization of the technical setup to avoid aggregation and unspecific adsorption to the surface (47, 48). When BPI_N_ labeled with NT647 was tested by MST for its affinity to LPS EC, it exhibited strong binding with a K_D_ in a low nanomolar range (Figure 3A). Binding of the corresponding rBPI NT647 to LPS EC was comparable to that measured for BPI_N(A)_ NT647, although a tendency toward lower affinity for rBPI was detected (K_D_ 17.8 ± 5.3 nM, Figure 3B). Since the MW of LPS EC is heterogenic due to natural variability in the length of the polysaccharide (34), the K_D_ value of rBPI to LPS was controlled with the defined rough-type LPS EC BL21 (MW 3.77 kDa). This K_D_ tended to be higher but was not decidedly different (72.6 ± 15.1 nM) compared to LPS EC (Figure 3B).

Next, the interaction between BPI and bLPs was assessed. Pam_3_CSK_4_ bound to BPI_N(A)_ NT647 with a high affinity (Figure 3C). Although working under strict low endotoxin conditions, we wanted to exclude contamination with LPS. Therefore, Pam_3_CSK_4_, covalently labeled with fluorescein, was tested for binding to unlabeled BPI_N(A)_ (Figure 3D). Indeed, the K_D_ was similar, or even lower, compared to that observed for binding of Pam_3_CSK_4_ to BPI_N(A)_ NT647, proving that Pam_3_CSK_4_ Fluorescein itself binds to BPI. (*R*)-Pam_3_CSK_4_ also bound to rBPI NT647 (K_D_ 161.5 ± 23.5 nM, Figure 3B). Nevertheless, as seen for LPS EC, the affinity was not as strong as observed for BPI_N(A)_ NT647.

The interaction of a protein with its ligand may result either in stabilization of the protein, and consecutively in an increase in melting temperature (T_m_), or in destabilization with a decrease in T_m_. To further elucidate this hypothesis, NanoDSF was performed. When rBPI was incubated with different ligands, including LPS EC, (*R*)-Pam_3_CSK_4_, (*R*)-Pam_2_CSK_4_, and (*R*)-FSL-1, an impressive decrease in T_m_ was found (Figures 3E,F).

In addition to the solid phase BPI binding assay, we demonstrated interactions between BPI and synthetic bLPs using MST and NanoDSF. Affinity of BPI to (*R*)-Pam_3_CSK_4_ was within a nanomolar range, but lower than that toward LPS EC as determined by MST.

### Binding of BPI to bLPs Depends on Both the Pattern of Acylation and the Structure of the Residual Peptide

**Figure 4:**
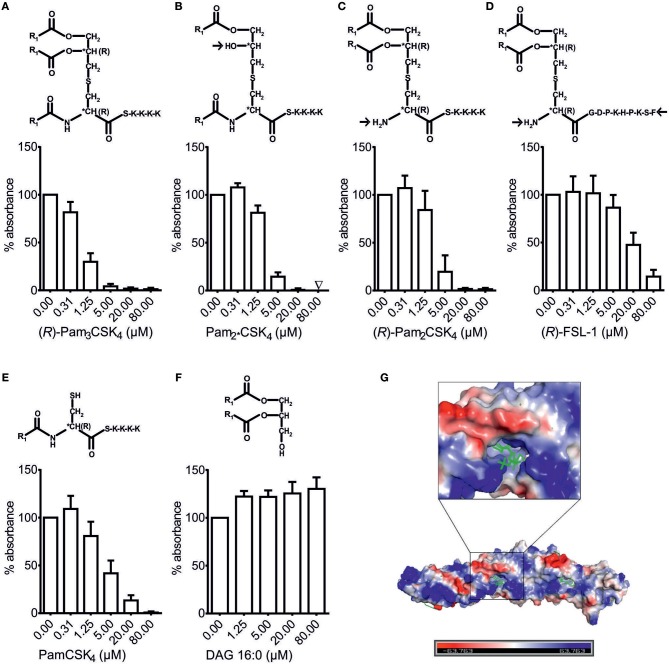
Structural requirements for binding of bLPs to BPI. BPI binding assay **(A–F)**. Lipopeptides with modified structure as well as DAG were tested for their potential to inhibit binding of rBPI to LPS biotin-coated wells **(A–F)**. Absorbance measured at 450 nm for wells with rBPI alone was set to 100% to ensure comparability between the different ligands. All results are shown as means ± SD of at least four biological replicates. R_1_ = CH_3_(CH_2_)_14_. Electrostatic charge surface of the BPI structure **(G)**. N-terminal lipid-binding pocket surrounded by differently charged areas is depicted with phosphatidylcholine as ligand.

Although LBP also binds bLPs (9, 10), the exact structural motifs for this interaction have not been determined yet. As shown in Figure 4, bLPs consist of a glycerol-core modified by two fatty acids and cysteine, leading to the diacyl-thioglycerol motif, which is crucial for TLR2-sensing of bLPs (4, 5). The cysteine is linked to a residual peptide at its C-terminus in all bLPs, and additionally to a fatty acid at its N-terminus in tri-acylated, but not in di-acylated bLPs (49). In order to investigate which structural motif of the bLPs is crucial for binding to BPI, variations of bLPs were compared to (*R*)-Pam_3_CSK_4_, which was the synthetic bLP with the highest affinity to BPI observed so far (Figure 4A). Deletion of the acyl-chain at the *O*-2*-*position of the thioglycerol core (Pam${}_{2^{*}}$CSK_4_) or at the amino-terminal cysteine of the residual peptide [(*R*)-Pam_2_CSK_4_], each resulted in a lower inhibitory potential (Figures 4B,C). Exchange of the residual peptide with a less positively charged sequence additionally impaired the affinity in this setting (Figure 4D). Moreover, the complete deletion of the glycerol-core with its two fatty acids, leaving the peptide with only one fatty acid (PamCSK_4_), was not enough to completely block the inhibitory potential (Figure 4E). In contrast, the glycerol-core (DAG) without a residual peptide showed no inhibitory binding to rBPI (Figure 4F). In contrast to the negatively charged LPS, (*R*)-Pam_3_CSK_4_ is highly positively charged due to the lysine-rich residual peptide. Interestingly, when looking at the surface charge distribution pattern of BPI (Figure 4G), both positively and negatively charged areas can be found around its N-terminal lipid-binding pocket (24). Consequently, binding sites are provided for the hydrophilic portion of contrarily charged ligands. Thus, our results demonstrate that both, the number of acyl-chains as well as the residual peptide and its amino-acid composition, influence the binding affinity of bLPs to BPI.

### Binding of BPI to Gram-Positive Bacteria does not Depend Solely on bLPs but also on LTA

The bLPs are synthesized in several steps as reviewed by Nakayama et al. (49). The first enzyme Lgt transfers a diacylglyceryl to the sulfhydryl group of the cysteine of a pre-prolipoprotein, generating a thioether linkage. Although deficiency in Lgt does not result in a lack of the specific lipoproteins (36), the lipoproteins lack the diacyl-anchor, which is one of the structural motifs that facilitates the binding of BPI to bLPs as shown in Figure 4. The second enzyme Lsp subsequently cleaves the signal peptide at the N-terminus of the diacylglyceryl cysteine of the prolipoprotein. Similarly, deficiency in Lsp does not result in the absence of the specific lipoproteins but in their specific modification (36). Since the signal peptide is not cleaved, the secondary structure of the lipoproteins is changed and the further coupling of a third acyl-chain to the central cysteine is prohibited. This acyl-chain is also important for the binding of BPI to bLPs (Figure 4).

**Figure 5:**
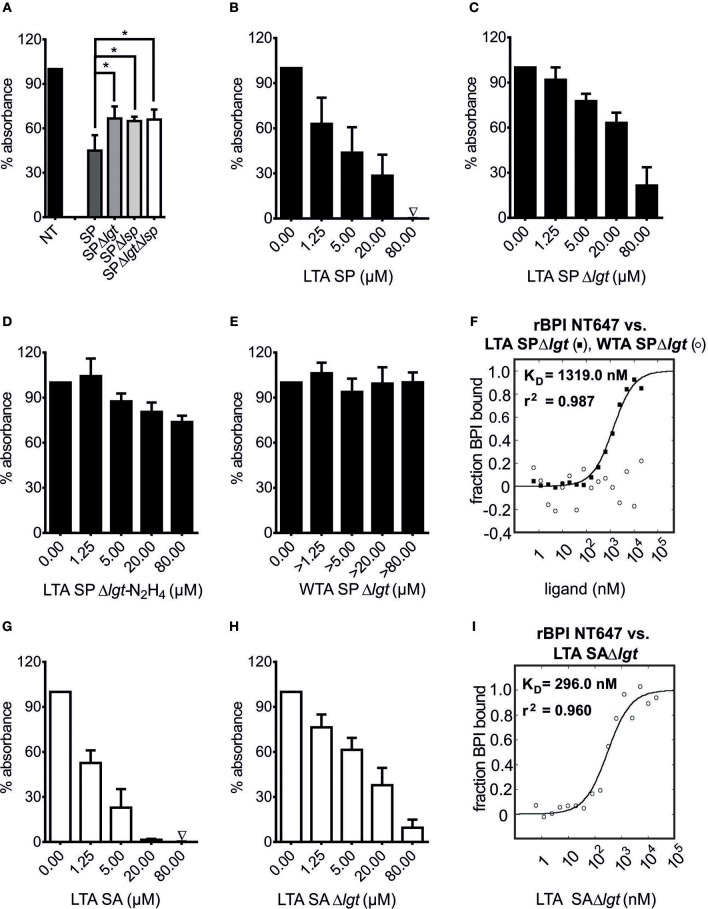
LTA is an additional ligand of BPI in Gram-positive bacteria. BPI binding assay **(A**–**E**, **G**,**H)**. MST binding assay **(F,I)**. Lysates of *S. pneumoniae* D39Δ*cps*, D39Δ*cps*Δ*lgt* (SPΔ*lgt*), D39Δ*cps*Δ*lsp* (SPΔ*lsp*) and D39Δ*cps*Δ*lgt*Δ*lsp* (SPΔ*lgt*Δ*lsp*; 50 μg/ml) were incubated with rBPI to evaluate their potential to inhibit the binding to LPS biotin-coated wells (**A**, NT: not treated). Statistics for comparison were performed with the unpaired Student's *t*-test (*p*-value *p = 0.004). LTA SP and LTA SA were tested for their potential to inhibit the binding of rBPI to LPS biotin-coated wells **(B,G)**. To correct for the effects of contaminating bLPs, LTAs of the corresponding Δ*lgt* strains were tested **(C,H)**. Data for LTA SPΔ*lgt*-N_2_H_4_ as well as WTA SPΔ*lgt* are shown **(D,E)**. Absorbance measured at 450 nm for wells with rBPI alone was set to 100% to ensure comparability between the different ligands. Results are shown as means ± SD of three **(B**–**E**, **G**,**H)** or four **(A)** biological replicates. rBPI NT647 was monitored in MST when incubated with increasing concentrations of LTA SPΔ*lgt* and WTA SPΔ*lgt* **(F)** as well as increasing concentrations of LTA SAΔ*lgt* **(I)**. K_D_ and *r*^2^ values are indicated for LTA SPΔ*lgt* and LTA SAΔ*lgt* **(F,I)**. Data represent the mean of three technical replicates **(F,I)**.

To test the hypothesis that bLPs are the ligands of BPI in Gram-positive bacteria, mutants of *S. pneumoniae* D39Δ*cps* lacking the *lgt*- and/or *lsp*-gene (36) were tested with the BPI binding assay (Figure 5A). When whole cell lysates of *S. pneumoniae* D39Δ*cps*, Δ*cps*Δ*lgt*, Δ*cps*Δ*lsp*, or Δ*cps*Δ*lgt*Δ*lsp* were tested, each inhibited binding of rBPI to LPS biotin-coated wells. However, inhibition was less pronounced for the Lgt-, Lsp-, or double-deficient strains in the intermediate concentration (*p* = 0.004, *n* = 4, unpaired Student's *t*-test).

Concerning additional binding partners of BPI, we examined LTA, which is anchored to the bacterial cell membrane via a lipophilic anchor. As exemplified in Figure S3, the common motif for all LTAs is a DAG, which is further substituted with a glycosyl moiety at the *O*-3 position. These glycosyl moieties, as well as the structures of present repeating units [polyglycerolphosphate (type I), complex glycosyl-glycerol-phosphate (type II + III), glycosyl-ribitol-phosphate (type IV) or glycosyl-phosphate (type V) containing polymers] are highly variable between different species of Gram-positive bacteria (50). As representative samples, we tested LTA preparations of two organisms with completely different LTA structures, *S. pneumoniae* [type IV LTA; (14)] and *S. aureus* [type I LTA; (39)], for their BPI binding potential. In order to ensure the purity of LTAs, not only LTA SP and LTA SA, but also LTA of the corresponding Lgt-deficient mutants (LTA SPΔ*lgt*, LTA SAΔ*lgt*) were tested. All competed for the binding of rBPI to LPS biotin as determined by the BPI binding assay (Figures 5B,C,G,H). This inhibition was less pronounced in experiments performed with LTA purified from Lgt-deficient strains, showing that, analogously to the synthetic bLPs, naturally occurring bLP variants are indeed binding partners of BPI in *S. pneumoniae* D39Δ*cps* as well as *S. aureus* 113. The binding potential to rBPI tended to be higher for LTA SAΔ*lgt* than LTA SPΔ*lgt* (Figures 5C,H). In accordance with this observation, MST also showed slightly superior binding of LTA SAΔ*lgt* compared to LTA SPΔ*lgt* (Figures 5F,I). Thus, LTAs from different bacterial genera exhibit differences in their affinity to BPI.

Next the structural motif necessary for binding of BPI to LTA was determined. As we have shown for bLPs, the binding to BPI was dependent on the acyl-chains. To evaluate the role of the diacyl-anchor of LTA, LTA SPΔ*lgt* was treated with anhydrous hydrazine for removal of the acyl moieties from the anchor (LTA SPΔ*lgt*-N_2_H_4_, depicted in Figure S3B). As shown in Figure 5D, competition in binding to rBPI of LTA SPΔ*lgt*-N_2_H_4_ was markedly reduced compared to LTA SPΔ*lgt*. As an independent approach, WTA of *S. pneumoniae* was used. *S. pneumoniae* WTA shares an identical chemical structure within its repeating units to LTA, but due to covalent attachment to the peptidoglycan (PGN), it lacks the diacyl-anchor that anchors LTA to the cytoplasmic membrane (14, 51). To mimic the natural situation as closely as possible, but still removing all other potential bioactive cell wall components, we used the pneumococcal PGN-WTA complex after treatment with the pneumococcal amidase LytA for our investigations. In this complex, pneumococcal WTAs are intact and bound to polymeric PGN-derived [MurNAc-GlcNAc]_x_-chains that are devoid of peptides (38). As shown in Figure 5E, this WTA SPΔ*lgt* did not compete with LPS biotin for binding to rBPI. It should be noted that the MW of PGN-WTA cannot be determined exactly. Thus, as stated in the methods, concentrations exceeding that of LTA were tested. Correspondingly, WTA SPΔ*lgt* also showed no binding to rBPI NT647 in MST (Figure 5F).

In summary, we describe two components originating from Gram-positive bacteria as new ligands of BPI including not only synthetic, but also naturally occurring bLPs and LTA. Interaction of LTA with BPI depends on its diacyl-anchor.

### BPI Enhances the Response to bLPs in PBMCs

**Figure 6:**
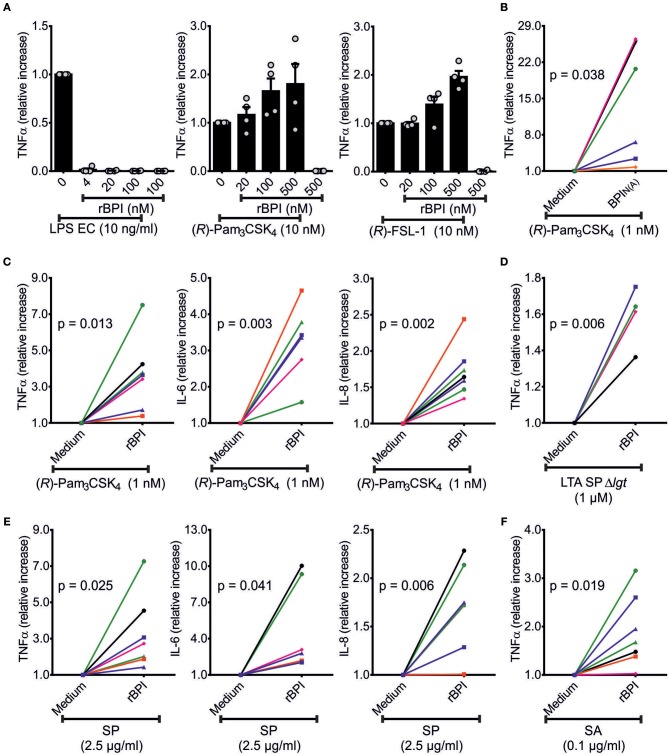
Influence of BPI on TNFα secretion of PBMCs in response to bLPs and lysates of *S. pneumoniae* D39Δ*cps*. TNFα-ELISA of the supernatants of PBMCs stimulated with TLR-ligands [LPS EC, (*R*)-Pam_3_CSK_4_ or (*R*)-FSL-1] ± rBPI at the indicated concentrations for 18 h **(A)**. Independent titration experiments performed in four different donors are indicated as relative increase compared to cytokine secretion with TLR ligand alone **(A)**. The relative increase in TNFα, IL-6, and IL-8 in the supernatants of PBMCs stimulated with (*R*)-Pam_3_CSK_4_ ± rBPI is represented **(C)**. One donor (•) is not shown in **(C)** for stimulation since IL-6 was beyond the linear range of the ELISA. The relative increase in TNFα in the supernatants of PBMCs stimulated with (*R*)-Pam_3_CSK_4_ ± BPI_N(A)_ or LTA SP ± rBPI is summarized **(B,D)**. PBMCs were stimulated with lysates of *S. pneumoniae* D39Δ*cps* and *S. aureus* 113 and the relative change in secreted TNFα, IL-6, and IL-8 caused by the addition of BPI is shown **(E,F)**. Results of independent stimulations of PBMCs of four **(A,D)** or seven **(B,C,E,F)** different donors are shown. Each symbol represents an individual donor **(B–F)**. Unless otherwise indicated, the BPI concentration used was 500 nM. Statistics for comparison of the relative cytokine secretion ± BPI were performed with the paired Student's *t*-test (*p*-values are indicated, **B**–**F**). Results are shown as means ± SEM **(A)**.

Next we investigated whether BPI is able to neutralize or indeed enhances the stimulatory potential of the newly described ligands. First, we optimized the PBMC cell culture conditions in order to achieve optimal neutralization of LPS by rBPI (Figure 6A). When bLPs were added to the PBMCs together with rBPI, no neutralization effect could be observed. In contrast, when the rBPI concentration was increased, TNFα secretion was gradually enhanced for both tri-acylated [(*R*)-Pam_3_CSK_4_] and di-acylated [(*R*)-FSL-1] bLPs (Figure 6A). This enhancement was not only detected for TNFα, but also for IL-6 and IL-8 with PMBCs of seven different donors (Figure 6C, Figures S4A,B). BPI derived from neutrophils had an even more pronounced function compared with the recombinant protein (Figure 6B, Figure S4C). As tested for (*R*)-Pam_3_CSK_4_, the enhancing effect was more efficient when lower concentrations of the stimulant were used as seen for both BPI preparations (10 nM vs. 1 nM). Since bLPs were shown to be bound by rBPI in the lysate of *S. pneumoniae* D39Δ*cps* (Figure 5A), we tested whether rBPI would influence the stimulatory potential of this lysate. Strikingly, PBMCs responded with a remarkable increase in TNFα, IL-6, and IL-8 secretion in the presence of rBPI in seven different donors (Figure 6E, Figure S5A). As expected, the corresponding lysate of the Δ*lgt*Δ*lsp*-mutant ± rBPI stimulated only weakly (Figure S5A). When lysates of *S. aureus* 113 were tested on PBMCs, TNFα, IL-6, and IL-8 secretion was also increased by addition of rBPI (Figure 6F, Figure S5C). Three independent lysates of *S. pneumoniae* D39Δ*cps, S. pneumoniae* D39Δ*cps*Δ*lgt*Δ*lsp* and *S. aureus* 113 were tested with comparable results (Figures S5B,D). As we have published previously (14), LTA SAΔ*lgt* did not show stimulation of TNFα secretion, and LTA SPΔ*lgt* stimulated TNFα secretion only at high concentrations (Figure S6). BPI incubated with LTA SPΔ*lgt* at the indicated concentrations increased secretion of TNFα compared to LTA SPΔ*lgt* alone (Figure 6D). LBP depends on CD14 for the transfer of LPS or bLPs to the corresponding TLRs (6, 9, 29). However, expression of TLR2 and CD14 in HEK293T cells is not sufficient to enhance immune stimulation of bLPs by BPI (Figure S7).

In summary, BPI enhances the immune response toward Gram-positive ligands in PBMCs when synthetic bLPs, LTA SPΔ*lgt* as well as lysates of Gram-positive bacteria are used.

## Discussion

BPI is a constitutively highly expressed protein in human neutrophils (30, 52), and has been recognized as a bactericidal protein toward Gram-negative bacteria (20, 30). Concerning the pathophysiological reason for the elevation of BPI in Gram-positive meningitis, direct bacterial killing seems unlikely, as this could not be shown for Gram-positive bacteria in previous studies (30, 31). However, rBPI_21_ protects mice against *S. pneumoniae* infection after intranasal application, an effect possibly caused by interactions with bacterial ligands (53). Here, we defined bLPs and LTA of Gram-positive origin as new ligands of BPI.

The finding that bLPs and LTAs compete with LPS for binding to BPI indicates that all three types of ligands apparently use a common binding site. This site is probably the N-terminal lipid-binding pocket described by Beamer et al. which is able to incorporate hydrophobic acyl-chains as shown for phosphatidylcholine (24). The dependency on the acyl-chains in BPI binding to bLPs and LTAs is in line with their important role for the binding of BPI to LPS (54). BPI binds to a variety of LPS preparations of different bacterial origin, all containing negatively charged anionic groups (54). Initially, it was surprising that the positive charge of bLPs favored high affinity binding to BPI, but this contradiction could be explained by the negatively and positively charged areas surrounding the N-terminal lipid-binding pocket of BPI. These opposing areas likely facilitate the stabilization of the hydrophobic interaction of different ligands. According to our data, BPI correlates with pro-inflammatory markers like TNFα in CSF of patients with pneumococcal meningitis. Importantly, binding of BPI to bLPs and LTA SP augmented the stimulatory potential in cell culture experiments. For bLPs, BPI enhanced the potential of TLR2/1-specific bLPs, as exemplified for (*R*)-Pam_3_CSK_4_, TLR2/6-specific bLPs, as exemplified for (*R*)-FSL-1, and natural bLPs, as exemplified by comparison of lysates of Lgt-deficient with the corresponding wild-type strain of *S. pneumoniae*. These effects are surprising and in contrast to the known neutralization of the TLR4-ligand LPS. In principle, the immune-enhancing capacity toward bLPs seems to be conserved in BPI and LBP. Whereas, LBP depends on CD14 for its function (6, 9, 29), CD14 is not essential for binding of BPI to PBMCs (27, 28). In accordance, CD14 was not sufficient to enable the enhancing effect of BPI in HEK293T cells transfected with a TLR2 expressing vector. Although the exact mechanism used by BPI still has to be elucidated, our data indicate a conservation of the lipid transfer function as known for other members of the TULIP family such as LBP or CETP. In this aspect, the conformational change of BPI upon exposure to LPS EC and bLPs as found in NanoDSF is congruent with data for CETP, which increases the diameter of its intra-molecule tunnel when bound to its specific binding partners, and consequently promotes the transfer of cholesteryl esters (55). A corresponding transport function might be operative for bLPs. The K_D_ value of 5.2 nM found in our study for BPI from neutrophilic source and LPS EC was in line with the published K_D_ of 4.1 nM for lipid A of *E. coli* J5 (56). Of note, the overall affinity of different bLPs to BPI tended to be lower than that seen for LPS. This difference seems important for either neutralization in case of high affinity or enhancement of the cell stimulatory activity when the affinity is lower. Thus, higher affinity, e.g. caused by additional interaction with the cationic tip, or other unknown factors, might omit transfer of LPS. In contrast to LPS, non-immune stimulating substances like synthetic bLPs lacking dihydroxypropylcystein such as PamCSK_4_ or 2-acyl-chains in position C1 and C2 of the glycerol such as Pam${}_{2^{*}}$CSK_4_, might be transferred by BPI but will not become immune stimulatory as they lack TLR2-stimulatory potential (57). In conclusion, we postulate that BPI is promiscuous in binding and transferring bacterial derived ligands like glycolipids or lipopeptides, but specificity with regard to the induced pro-inflammatory response is determined by the respective recognizing receptor.

Surprisingly and in contrast to BPI, we found no correlation between LBP and cytokines in the CSF samples. Although BPI and LBP were described as antagonists with respect to their action toward LPS (21), LBP can not only increase (11), but also antagonizes the stimulatory potential of LPS when LBP concentrations are high (58, 59). This could be true for bLPs as well. Thus, the function of both proteins seems to be complementary and not merely antagonistic. Since BPI is secreted locally from neutrophils and epithelial cells (23, 60, 61), and LBP is instead released mainly systemically from hepatocytes (62, 63), their function is possibly adjusted to different sites of action.

As shown previously, BPI lacks a major neutralizing effect on endotoxin within meningococcal bacterial outer membrane vesicles in monocyte-derived dendritic cells (64). In line with our data, this might also be a result of the immune-enhancing potential of BPI toward bLPs, therefore masking the neutralizing capacity toward the endotoxin. In human neutrophils, the combination of BPI and *E. coli*, but not of BPI and LPS alone, was found to enhance degranulation in neutrophils (26), which likewise could be caused by the interaction between BPI and bLPs present in *E. coli*. Recently, a significant enhanced regeneration of bone marrow cells by repeated application of high doses of rBPI_21_ to mice after total body irradiation was described (65). Simultaneously, an increase of pro-inflammatory cytokines, such as TNFα and IL-6, was found in plasma. The underlying mechanism for this finding remained unclear, albeit effects of rBPI_21_ on endothelium and pericytes in the hematopoietic microenvironment were suggested. The pro-inflammatory potential of BPI toward TLR2-ligands is a process possibly involved. Irradiation causes damage to the epithelial barrier of the gut and consequentially transition of the intestinal microbiota (66), which is able to promote myeloid differentiation to granulocyte and/or monocyte progenitors in the bone-marrow (67). Treatment of irradiated mice with a TLR2 agonist also results in accelerated hematopoiesis (68). BPI most likely enhances the TLR-mediated immune response caused by released bacteria after total body irradiation of mice. In a translational perspective, co-application of TLR2 ligands with BPI might even enhance effects of BPI on haematopoesis and therefore facilitate dose reduction and future cost effective application to patients.

Compared to other antimicrobial peptides, BPI is unique since it neutralizes LPS with high efficiency on the one hand, and specifically enhances the inflammatory response to bLPs and LTA on the other. Based on these findings, BPI is a soluble pattern recognition molecule with opposing effects on different bacterial ligands, and therefore has the potential to adapt the immune response according to the class of bacteria encountered. Further investigations concerning the pro-inflammatory functions of BPI are needed as they will contribute to the principle understanding of innate immunity against bacterial infections.

## Author Contributions

LZ, JH, TS, and CE conducted the experiments. MW expressed rBPI. MT performed Luminex measurements. SH contributed mutant bacterial strains and bacterial material for LTA and WTA preparations. FW and NG contributed LTAs, WTA SPΔ*lgt*, and LPS EC BL21. SB wrote the main text of the manuscript. SB and AG conceived the experiments, analyzed the results, and oversaw the project. All authors discussed the experiments and reviewed the manuscript.

### Conflict of Interest Statement

The authors declare that the research was conducted in the absence of any commercial or financial relationships that could be construed as a potential conflict of interest.
