# Supplementary material for: Bactericidal/Permeability-Increasing Protein Is an Enhancer of Bacterial Lipoprotein Recognition
Source: Front Immunol. 2018 Dec 5;9:2768. doi: 10.3389/fimmu.2018.02768 (PMC6293271; doi:10.3389/fimmu.2018.02768)
Supplement: Supplementary file 1 [file Data_Sheet_1.pdf]

Supplementary Material

Bactericidal/Permeability-Increasing Protein Is an Enhancer of Bacterial Lipoprotein Recognition

Sigrid Bülow, Lisa Zeller, Maren Werner, Martina Toelge, Jonas Holzinger, Clemens Entzian, Thomas Schubert, Franziska Waldow, Nicolas Gisch, Sven Hammerschmidt, André Gessner*

*** Correspondence:** André Gessner: andre.gessner@klinik.uni-regensburg.de

Supplementary Figures

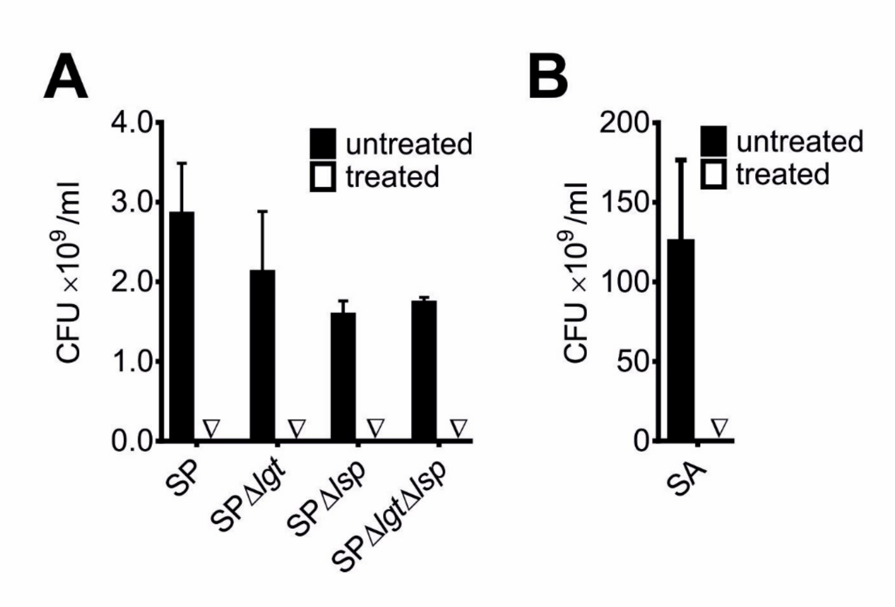

Figure S1

Characterization of bacterial lysates. Bacterial lysates of *S. pneumoniae* D39Δ*cps* strains (**A**) and *S. aureus* 113 (**B**) were tested for viability after heat-inactivation.

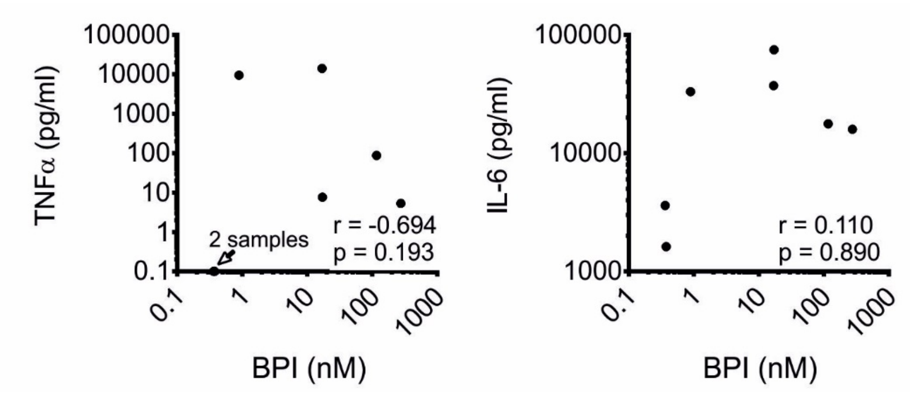

Figure S2

Correlation of BPI with pro-inflammatory markers in Gram-negative meningitis. Correlation of BPI with TNFα and IL-6 in CSF of patients with bacterial meningitis caused by NM (n = 7) is depicted. Correlation was analyzed using Pearson's correlation (r- and p-values are indicated). Logarithmic values were used for statistical testing.

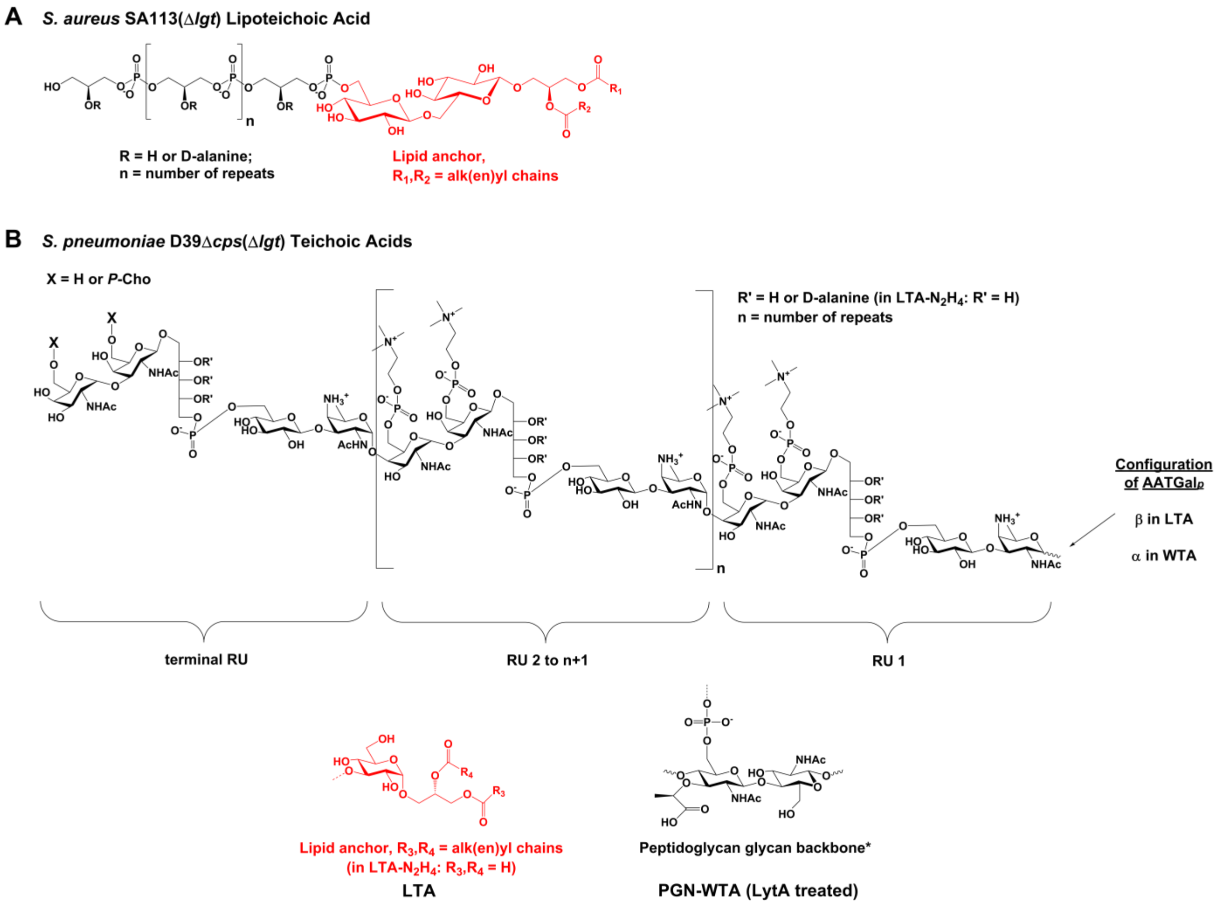

Figure S3

Chemical structures of *S. aureus* LTA, *S. pneumoniae* LTA, and *S. pneumoniae* PGN-WTA preparations used in this study. *S. aureus* LTA contains β-D-Glc*p*-(1→6)-α-D-Glc*p*-(1→3)-diacylglycerol as lipid anchor (colored in red), which is further substituted with a poly-glycerolphosphate chain. The average number of glycerolphosphate repeats in the used preparations is n = 16 (WT) or n = 18 (Δ*lgt*) with a degree of alanylation of appr. 30%, as determined by ^{1}H NMR according to a published procedure (39) (**A**). Pneumococcal teichoic acid chains are composed of pseudo-pentasaccharide (AATGal*p* – Glc*p* – Rib-ol-5-*P* – 6-*O*-*P*-Cho-Gal*p*NAc – 6-*O*-*P*-Cho-Gal*p*NAc) repeating units (RU), the terminal RU can occur with or without 6-*O*-*P*-Cho-substitution. In LTA, the first RU is β-1-linked to the lipid anchor (α-D-Glc*p*-(1→3)-diacylglycerol), which is shown in its native form and after de-*O*-acylation with N_2H_4 (both colored in red). In WTA, the respective linkage to the peptidoglycan (PGN) backbone has an α-configuration. All other RUs are α-glycosidically linked to the previous one. The chain length of pneumococcal teichoic acids ranges between 4 and 8 RU, with molecules with 6 RU as major species (14, 40). In the used PGN-WTA preparation all peptides have been removed by LytA treatment (**B**).

* The exact chemical composition of this pneumococcal PGN-WTA macromolecule is not known, especially with regard to the ratio of [MurNAc-GlcNAc]-units and WTA chains.

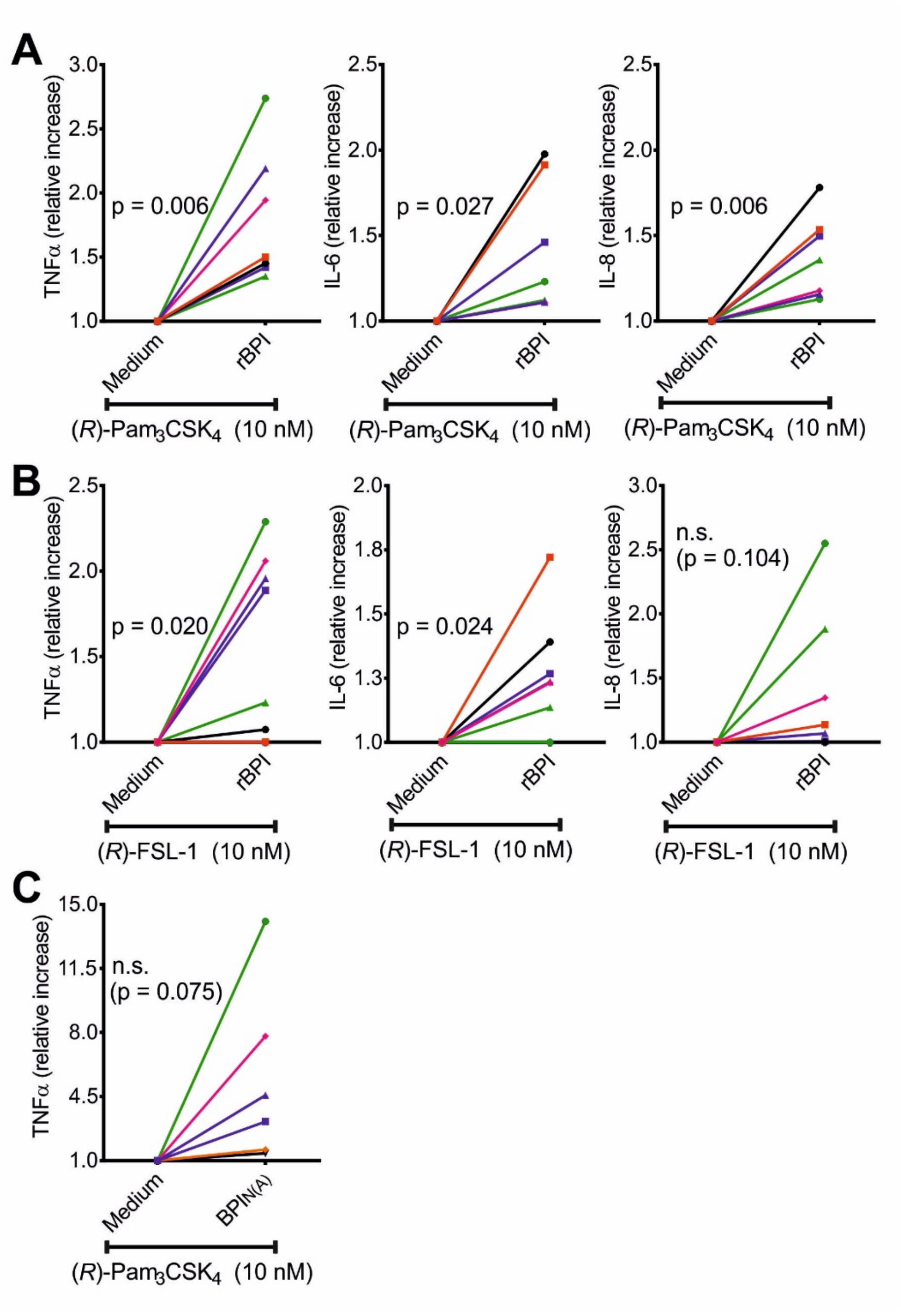

Figure S4

Influence of BPI on TNFα secretion of PBMCs in response to bLPs. The relative increase in TNFα, IL-6 and IL-8 in the supernatants of PBMCs stimulated for 18 h with (*R*)-Pam_3CSK_4 or (*R*)-FSL-1 at the indicated concentrations ± rBPI is represented (**A**, **B**). Independent stimulations of PBMCs of seven different donors are summarized. The relative increase in TNFα in the supernatants of PBMCs stimulated for 18 h with (*R*)-Pam_3CSK_4 ± $BPI_{N(A)}$ is represented (**C**). The results of independent stimulations of PBMCs of six different donors are summarized. The BPI concentration used here was 500 nM. Statistics for comparison of the relative cytokine secretion ± rBPI were performed with the paired Student's *t* test (p-values are indicated).

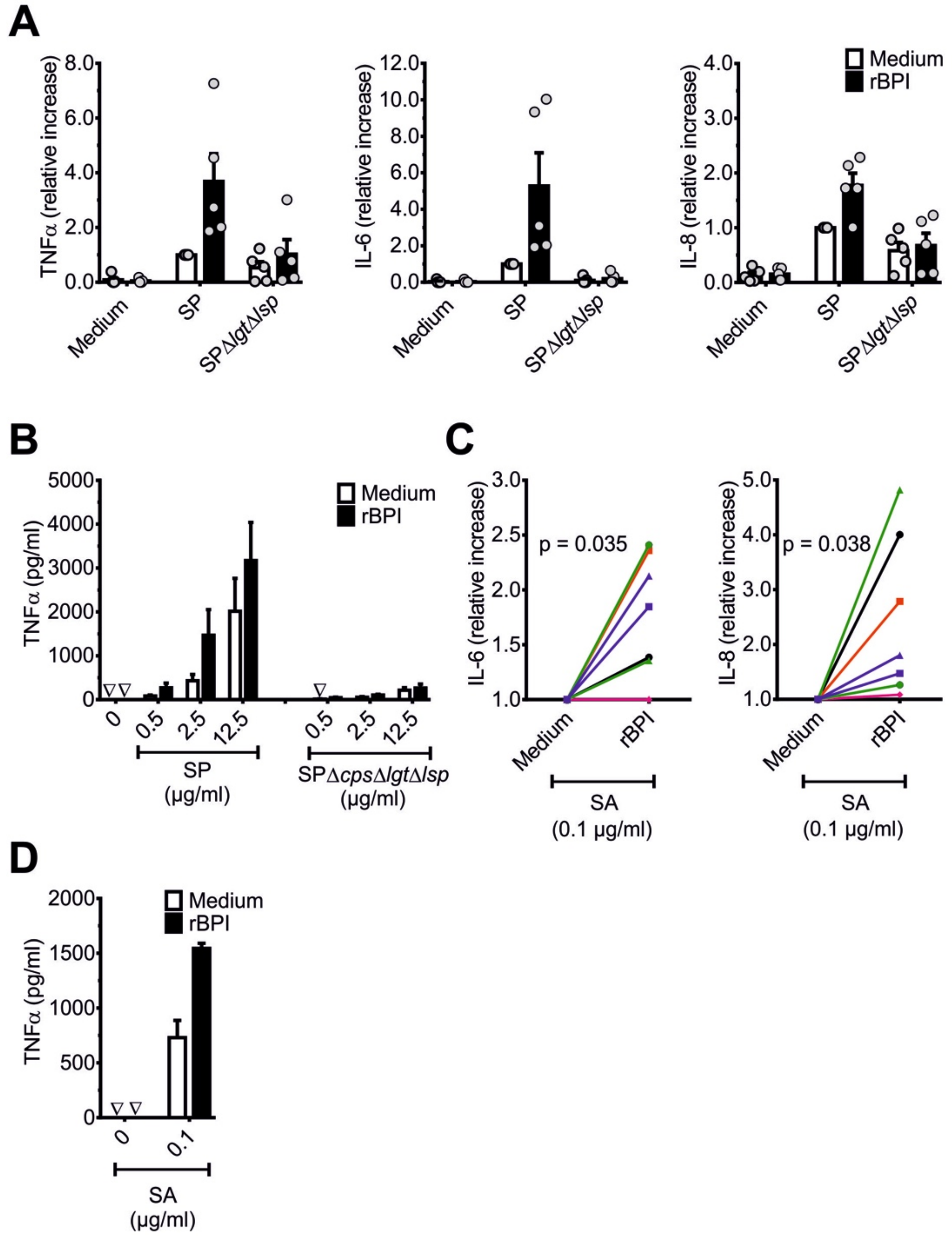

Figure S5

Influence of BPI on the response of PBMCs to lysates of Gram-positive bacteria. PBMCs five different donors were stimulated with lysates of *S. pneumoniae* D39Δ*cps* (SP) and *S. pneumoniae* D39Δ*cps*Δ*lgt*Δ*lsp* (SPΔ*lgt*Δ*lsp*) at a concentrations of 2.5 µg/ml ± rBPI. TNFα**,** IL-6- and IL-8-ELISA are depicted as relative increase compared to cytokine secretion with *S. pneumoniae* D39Δ*cps* alone (**A**). The relative change in secreted IL-6 and IL-8 caused by the addition of BPI to lysates of *S. aureus* 113 (SA) is shown for independent stimulations of PBMCs of seven different donors (**C**). The rBPI concentration used was 500 nM. Each symbol represents an individual donor. Three independent lysates of *S. pneumoniae* D39Δ*cps*, *S. pneumoniae* D39Δ*cps*Δ*lgt*Δ*lsp* and *S. aureus* 113 were tested in one donor with comparable results (**B**, **D**). Statistics for comparisons of the relative cytokine secretion ± rBPI were performed with the paired Student's *t* test (p-values are indicated). Results are shown as means ± SEM (**A**, **B**, **D**).

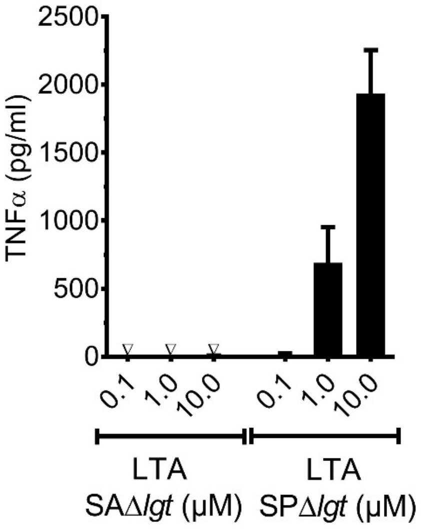

Figure S6

TNFα secretion of PBMCs in response to LTA of *S. pneumoniae* D39Δ*cps*Δ*lgt* (SPΔ*lgt*) and *S. aureus* 113 (SPΔ*lgt*). TNFα-ELISA of the supernatants of PBMCs stimulated with LTA SAΔ*lgt* and LTA SPΔ*lgt* at the indicated concentrations for 18 h are shown. Results of independent stimulations of PBMCs of five (LTA SAΔ*lgt*) and six (LTA SPΔ*lgt*) different donors are summarized as means ± SEM.

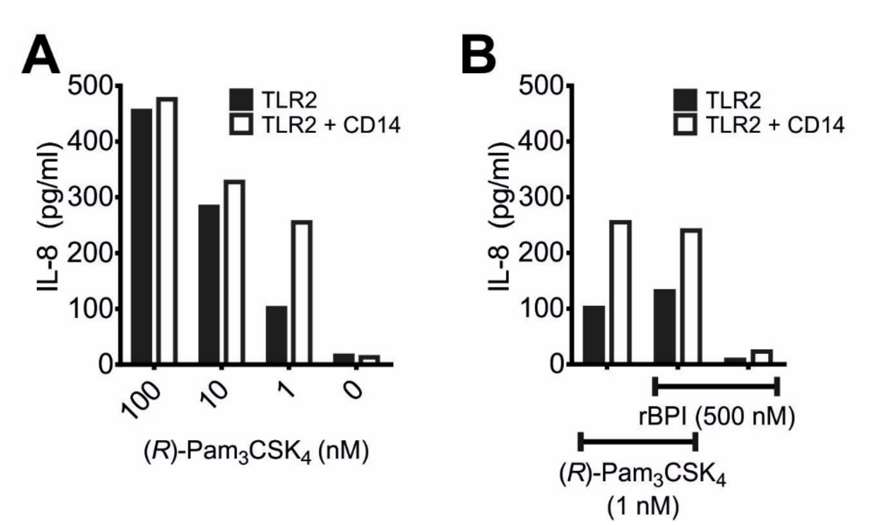

Figure S7

CD14 was not sufficient to enable the enhancing effect of BPI. HEK293T cells were transfected with plasmids encoding TLR2 and/ or CD14 and stimulated with increasing concentrations of (*R*)-Pam_3CSK_4 (**A**). Stimulation of (*R*)-Pam_3CSK_4 ± BPI is shown in (**B**). One representative result of two independent experiments is indicated (**A, B**).
